# Explainable AI in educational data mining: LightGBM and SHAP for enhancing learner performance prediction

**DOI:** 10.3389/frai.2026.1954446

**Published:** 2026-10-09

**Authors:** Zakaria Alj, Anas Bouayad

**Affiliations:** 1 Department of Economics, Mohammed V University, Faculty of Juridical Economic and Social Sciences, Souissi, Morocco; 2 Department of Computer Sciences, Universite Sidi Mohamed Ben Abdellah Faculte des Sciences Dhar El Mahraz, Fes, Morocco

**Keywords:** adaptive learning system, educational data mining, explainable AI, learner performance prediction, LightGBM

## Abstract

Accurate prediction of learner performance is essential for adaptive educational systems and personalized learning. However, the integration of advanced gradient-boosting models with established educational frameworks remains underexplored, particularly when predictive performance and interpretability are considered jointly. This study evaluates Light Gradient Boosting Machine (LightGBM) within three established learner-modeling frameworks—Item Response Theory (IRT), Performance Factor Analysis (PFA), and Dynamic Aspects of Student Skill with History (DAS3H)—across seven real-world educational datasets. LightGBM is compared with traditional logistic regression using the Area Under the Receiver Operating Characteristic Curve (AUC) and accuracy as evaluation metrics. To further assess model interpretability, SHapley Additive exPlanations (SHAP) are employed to identify the contributions of user-, item-, and skill-level features to learner performance predictions. Results show that LightGBM generally improves predictive performance compared with logistic regression, particularly in capturing nonlinear relationships, temporal patterns, and skill-dependent learner behaviors. The SHAP analysis further provides insights into the relative importance of different feature types, enhancing the interpretability of the predictive models. These findings demonstrate that integrating LightGBM with established learner-modeling frameworks can provide a more accurate and interpretable approach to learner performance prediction. The novelty of this study lies in combining LightGBM with IRT, PFA, and DAS3H, together with SHAP-based explainability, across multiple educational datasets, offering a comprehensive framework for accurate and interpretable learner modeling in adaptive and personalized learning environments.

## 1 Introduction

Educational systems have embraced data-driven technologies to transform learning experiences, offering personalized pathways and adaptive interventions tailored to individual learner needs. Predicting learner performance plays a pivotal role in this transformation, facilitating real-time feedback, targeted interventions, and enhanced educational outcomes. However, achieving accurate and interpretable predictions within the complex dynamics of educational data remains a persistent challenge (Zakaria et al., 2024).

Traditional models such as Bayesian Knowledge Tracing (BKT) (Corbett and Anderson, 1994) and logistic regression-based approaches, including Item Response Theory (IRT) (De Boeck, 2004) and Performance Factor Analysis (PFA) (Pavlik et al., 2009), have provided foundational methods for learner performance prediction. While these methods excel in interpretability and simplicity, their assumptions often fail to capture the nonlinear and temporal interactions inherent in real-world educational data. Recent advancements, such as Dynamic Aspects of Student Skill with History (DAS3H) (Choffin et al., 2019), have addressed some of these limitations by incorporating temporal and skill-specific features, yet challenges remain in achieving optimal predictive accuracy.

Machine learning techniques, particularly gradient boosting algorithms like Light Gradient Boosting Machine (LightGBM), have shown significant promise in addressing these limitations. LightGBM’s ability to handle sparse, sequential, and high-dimensional data positions it as a state-of-the-art tool for educational contexts (Ke et al., 2017). Recent studies have demonstrated its superior performance in predicting student outcomes (Hamim et al., 2022). However, its application in integrating domain-specific educational models, such as IRT, PFA, and DAS3H, remains underexplored. This gap represents a critical opportunity to leverage LightGBM’s predictive strengths while contextualizing it within well-established educational frameworks.

A parallel challenge in educational data mining (EDM) is the interpretability of machine learning models. While models like LightGBM offer high predictive accuracy, their “black-box” nature limits their utility in educational settings where understanding model predictions is essential for actionable insights. Explainable AI (XAI) methods, particularly SHapley Additive exPlanations (SHAP), address this challenge by providing a transparent mechanism for understanding feature contributions to model predictions (Lundberg and Lee, 2017). Despite its potential, the integration of SHAP with LightGBM for explaining learner performance prediction remains underexplored in the literature, especially when applied to diverse and complex educational datasets.

Recent developments in artificial intelligence further highlight the potential of adaptive and explainable learning approaches for complex prediction and decision-making tasks. Reinforcement learning, for example, has been employed to optimize multi-criteria decision processes by learning from interactions and feedback (Sahoo and Ghosh, 2026). Other recent studies have demonstrated the value of combining domain knowledge with deep learning and explainability, as illustrated by explainable physics-informed neural networks (Kayiran, 2026a), as well as explainable machine-learning frameworks using SHAP to improve transparency in complex predictive pipelines (Demir, 2026). In higher education, AI integration is also receiving increasing attention, with recent research emphasizing both its potential benefits and the operational, ethical, and governance challenges associated with its adoption (Ghaly, 2026). Although these approaches have been investigated across different application domains, they illustrate broader methodological trends toward adaptive, data-driven, and explainable AI that could also benefit learner performance prediction.

This study bridges these gaps by integrating LightGBM with SHAP to advance learner performance prediction and interpretability. Specifically, this work contributes to the literature in two key ways. First, it evaluates LightGBM’s performance against traditional logistic regression-based models (IRT, PFA, and DAS3H) across seven real-world datasets. Second, it employs SHAP to provide interpretable insights into feature importance, enabling educators to understand the drivers behind model predictions and make informed, data-driven decisions. These contributions advance the state-of-the-art in educational data mining, combining predictive accuracy with transparency to enhance the practical utility of predictive models in intelligent tutoring systems (ITS).

The remainder of this paper is organized as follows: Section 2 provides an overview of related work in learner performance prediction and machine learning interpretability in education. Section 3 discusses traditional logistic regression-based models, highlighting their strengths and limitations. Section 4 introduces LightGBM and its methodological advancements. Section 5 delves into explainability in educational data mining, focusing on the role of XAI and SHAP. Section 6 outlines the research methodology, including datasets, preprocessing, and experimental design. Section 7 presents the results, comparing the predictive and interpretive performance of LightGBM and traditional models. Section 8 discusses the implications of the findings. Section 9 presents future research directions, and Section 10 concludes the paper by summarizing its main contributions.

## 2 Related work

Machine learning models for learner performance prediction have evolved significantly, showcasing an increasing shift from traditional statistical methods like logistic regression to advanced ensemble and boosting techniques. Logistic regression, known for its simplicity and interpretability, has been widely applied in predicting learner outcomes, particularly in the context of educational data mining (EDM) and Intelligent Tutoring Systems (ITS) (Alj and Malki, 2023). Despite its foundational role, logistic regression faces inherent limitations in capturing dynamic and temporal learning behaviors, motivating the exploration of more sophisticated approaches (Wang et al., 2024).

Boosting frameworks have garnered attention for their ability to improve predictive accuracy and model complex dependencies. In recent studies, XGBoost has consistently demonstrated superior performance in educational prediction tasks. For example, Yan (2021) utilized XGBoost to analyze a macro-educational structured dataset, outperforming models such as Random Forest, Lasso, and Elastic Net. Similarly, Alsulami et al. (2023) reported an accuracy improvement from 0.75 to 0.77 through boosting techniques, emphasizing the model’s utility in integrating diverse interaction logs during the COVID-19 pandemic. These results align with findings by Asselman et al. (2023), who noted that XGBoost outperformed AdaBoost and Random Forest in learner prediction tasks.

Beyond XGBoost, LightGBM offers further advantages, including computational efficiency and scalability in high-dimensional and sparse datasets, attributes particularly relevant for educational applications (Ke et al., 2017). Its unique leaf-wise tree growth and feature handling techniques, such as Exclusive Feature Bundling (EFB) and Gradient-Based One-Side Sampling (GOSS), enhance its capability to model intricate learning trajectories (Chen and Guestrin, 2016). In the context of Performance Factor Analysis (PFA), where temporal and skill-specific attributes are critical, LightGBM’s ability to handle time-dependent features has shown promising results (Gervet et al., 2020).

Comparative studies have also explored the integration of boosting techniques with educational frameworks such as IRT, PFA, and DAS3H. For instance, Schmucker et al. (2021) improved the AUC of logistic regression models by introducing features derived from response logs and combining these with video and contextual data. While logistic regression excelled in interpretable scenarios, boosting models like LightGBM outperformed them in datasets with high temporal and interactional complexity. Similarly, Yeung and Yeung (2018) highlighted the importance of prediction-consistent regularization in enhancing deep knowledge tracing models, demonstrating the versatility of boosting techniques in capturing nuanced learner behaviors.

Ensemble approaches incorporating boosting have also proven effective in various studies. Fan et al. (2023) introduced P2catBoost, a dual particle swarm optimization-based boosting technique, achieving state-of-the-art results in predicting secondary school performance. These advancements underline the potential of boosting methods to generalize across diverse educational contexts, from skill acquisition to adaptive learning systems.

Despite these successes, a trade-off exists between predictive power and interpretability. Explainable AI (XAI) methods like SHAP have emerged as critical tools to address this challenge, offering insights into feature importance and model predictions (Lundberg and Lee, 2017). By integrating SHAP into boosting models, researchers have enhanced the transparency of predictions, enabling actionable insights for educators and system designers.

Recent research published in 2025 has further emphasized the shift toward more accurate and explainable approaches for student performance prediction. Kalita et al. (2025) proposed a Bi-LSTM framework combined with SHAP-based interpretation and reported improved predictive performance compared with several traditional machine-learning models, including LightGBM. Similarly, Johora et al. (2025) developed an explainable stacking ensemble model integrating SHAP and LIME to predict undergraduate academic performance, demonstrating the growing importance of combining predictive accuracy with transparency. More specifically, Abukader et al. (2025) combined LightGBM with metaheuristic hyperparameter optimization and SHAP-based learning analytics, showing that optimized gradient-boosting models can provide both strong predictive performance and interpretable feature-level insights. A systematic review by Choi et al. (2025) further confirmed the increasing adoption of XAI in student performance prediction, identifying SHAP as one of the most frequently used explanation techniques while highlighting the still limited use of explainability for individualized educational decision-making.

Recent studies in 2026 reinforce these trends while revealing persistent methodological limitations. Qiang et al. (2026) integrated educational domain knowledge with an explainable neural-network framework using SHAP and demonstrated that domain-informed modeling can enhance both interpretability and prediction. However, their framework primarily relied on static student characteristics, and the authors identified the incorporation of temporal and behavioral information as an important direction for future research. Likewise, Boujmiraz et al. (2026), in a systematic review of 72 studies on student performance prediction, reported that tree-based and neural-network approaches currently dominate the field, while the adoption of explainable AI remains comparatively limited. They also noted that relatively few studies explicitly connect predictive and explainable models with practical educational innovation and adaptive learning.

Despite these advances, several gaps remain. First, recent studies generally focus on generic academic, demographic, or behavioral predictors rather than systematically embedding advanced machine-learning models within established learner-modeling frameworks such as IRT, PFA, and DAS3H. Second, many studies evaluate their approaches within a single dataset or educational context, limiting evidence regarding cross-dataset robustness. Third, although SHAP and other XAI techniques are increasingly adopted, their integration with temporal, skill-specific, and learning-history features remains insufficiently explored. The present study addresses these gaps by systematically comparing LightGBM with logistic regression within the IRT, PFA, and DAS3H frameworks across seven real-world educational datasets. In addition, SHAP is employed to explain the contributions of user-, item-, skill-, and temporal-level features, thereby combining predictive performance, educationally grounded feature representations, and model interpretability.

This study builds on this body of work by comparing logistic regression with LightGBM in learner performance prediction. By employing boosting techniques and state-of-the-art evaluation metrics, the research seeks to address the limitations of traditional models and advance the understanding of temporal and dynamic learning behaviors in educational datasets.

## 3 Learner performance prediction with logistic regression

Educational Data Mining (EDM) seeks to uncover patterns and insights from learners’ interactions with educational systems, offering data-driven methods to enhance teaching and learning (Alj and Bouayad, 2024). A central challenge in this field is predicting student performance specifically, determining whether a student will answer a given question correctly. Accurate predictions not only support adaptive learning systems, such as Intelligent Tutoring Systems (ITS), but also provide valuable insights into individual learners’ strengths and weaknesses.

A variety of methods have been employed to tackle this prediction task, ranging from traditional statistical models to advanced machine learning techniques. Among these, logistic regression serves as a foundational approach due to its simplicity and interpretability.

In ITS and similar systems, a student’s interaction history is often represented as $\vec{x_{t}}=(x_{1},x_{2},\ldots ,x_{t})$ where each interaction $x_{t}=(q_{t},a_{t})$ consists of the question identifier $q_{t}$ and the correctness of the answer $a_{t}\in \{0,1\}.$

The logistic regression model estimates the probability of a correct response for the next question qt + 1 given historical interactions ${\vec{x}}_{1,t}$ as follows:

$$P(a_{t+1}=1q_{t+1},{\vec{x}}_{1:t})=S({\vec{w}}^{⊤}\Phi (q_{t+1},{\vec{x}}_{1:t}))$$

Where $S(z)=\frac{1}{1+e^{-z}}$ is the sigmoid function. Here, $\vec{w}$ represents a weight vector learned during training, and $\Phi (q_{t+1},{\vec{x}}_{1:t})$ is a feature mapping function that encodes the interactions. Logistic regression is intuitive and interpretable, making it a popular choice. However, it is inherently limited in capturing dynamic learning behaviors, such as time-dependent skill development or the impact of repeated practice. These limitations underscore the need for more sophisticated approaches that account for the temporal and evolving nature of learning (Pavlik et al., 2009).

To overcome the static assumptions of logistic regression, Item Response Theory (IRT) introduces a latent trait framework, focusing on the interaction between a student’s ability and the difficulty of the question. Unlike logistic regression, which relies heavily on feature engineering for encoding historical interactions, IRT models student performance by explicitly representing two key factors: the student’s ability and the inherent difficulty of a question. The probability of a correct response in IRT is given as:

$$P_{\mathrm{IRT}}(a_{t+1}=1q_{t+1})=S(A_{s}-D_{q_{t+1}})$$

Where $\left(A_{s}\right)$ is a latent trait capturing the learner’s overall skill, and $\left(D_{q_{t+1}}\right)$ Represents the inherent difficulty of question q . The sigmoid function ensures that the probability smoothly transitions based on the difference $\left(A_{s}-D_{q_{t+1}}\right)$ . While IRT provides an elegant approach to modeling student-question interactions, it assumes that a student’s ability remains constant over time. This static assumption limits its applicability in contexts where learning, practice, and forgetting play significant roles.

Recognizing the need to model dynamic learning processes, approaches such as Performance Factors Analysis (PFA) extend beyond IRT by incorporating a student’s historical interactions. PFA tracks successes and failures for individual knowledge components (KCs), offering a more granular representation of learning. For a question associated with a set of KCs, the probability of a correct response is computed as:

$$P\;(a_{t+1}=1q_{t+1})=S(\sum_{k\in \mathrm{KC}(q_{t+1})}(\beta_{k}+\gamma_{k}c_{s,k}+\rho_{k}f_{s,k}))$$

Where $\beta_{k}$ is the baseline difficulty of KC_k_, $\gamma_{k}$ is the learning gain associated with correct responses ( $c_{s,k}$ : the count of correct responses to KC_k_ by student s) and $\rho_{k}$ is the penalty associated with incorrect responses ( $f_{s,k}$ : the count of incorrect responses to KC_k_). While PFA successfully captures dynamic learning patterns, it does not account for question-specific difficulty or the effects of time on forgetting.

To address the limitations of PFA and IRT, the Dynamic Aspects of Student Skill with History (DAS3H) model integrates elements from both approaches while incorporating temporal features. DAS3H introduces time-dependent counters for correct and total attempts, grouped into predefined time windows. The model computes the probability of a correct response as:

$$P_{\mathrm{DAS}3H}(a_{t+1}=1q_{t+1},x_{1:t})=S(\begin{array}{l} (A_{s}-D_{q_{t+1}})+\sum_{k\in \mathrm{KC}(q_{t+1})}\beta_{k}\\ +\sum_{k\in \mathrm{KC}(q_{t+1})}\sum_{w=0}^{W-1}\\ \left(\begin{array}{l} \theta_{k,w}\log(1+c_{s,k,w})\\ -\phi_{k,w}\log(1+a_{s,k,w}) \end{array}\right) \end{array})$$

Where $c_{s,k,w}$ is the count of correct responses for KC_k_ by student s in time window w, $a_{s,k,w}$ is the count of total attempts for KC_k_ by student s in time window w, $\theta_{k,w}$ and $\phi_{k,w}$ are, respectively, coefficients capturing the impact of learning and forgetting. The logarithmic transformation ensures diminishing returns for repeated practice, while time-windowed counters enable DAS3H to model both short-term and long-term learning. By combining the strengths of IRT and PFA, DAS3H provides a more comprehensive approach to understanding student performance over time.

## 4 Light gradient boosting machine (LightGBM)

Light Gradient Boosting Machine (LightGBM), developed by Ke et al. (2017), represents a significant advancement in gradient boosting frameworks, offering a robust and efficient alternative to traditional models such as XGBoost (Chen and Guestrin, 2016). It is particularly advantageous in scenarios requiring computational efficiency and predictive accuracy across large-scale, sparse, and sequential datasets, such as those encountered in educational contexts like Item Response Theory (IRT), Performance Factor Analysis (PFA), and DAS3H.

Unlike XGBoost, which employs a level-wise growth strategy, LightGBM adopts a leaf-wise approach. This method grows the tree by expanding the leaf with the largest loss reduction at each step, enabling the model to achieve higher accuracy with fewer iterations. For predictive modeling tasks like DAS3H, which captures temporal dependencies and skill acquisition dynamics, the ability to prioritize splits that maximize gradient improvements is essential. This feature makes LightGBM especially effective in modeling the short- and long-term dependencies inherent in student learning trajectories (Ke et al., 2017).

LightGBM further distinguishes itself with its histogram-based decision tree learning, which discretizes continuous features into discrete bins. This innovation reduces memory usage and computational overhead without compromising model precision, an advantage particularly relevant for large datasets such as ASSISTments. In PFA, for instance, where evolving knowledge states and sparsity pose significant challenges, LightGBM’s efficient feature handling directly contributes to superior predictive power (Gervet et al., 2020).

Moreover, LightGBM incorporates two key innovations that enhance its performance over XGBoost: Exclusive Feature Bundling (EFB) and Gradient-Based One-Side Sampling (GOSS). EFB effectively handles high-dimensional data by bundling mutually exclusive features into single groups, reducing feature dimensionality without loss of interpretability. GOSS, on the other hand, prioritizes data points with larger gradients, ensuring the model focuses on hard-to-predict samples. These techniques are particularly advantageous in DAS3H, where variability introduced by temporal dynamics and forgetting curves demands efficient and scalable modeling techniques (Ke et al., 2017).

A further strength of LightGBM lies in its native handling of missing values and categorical data. Educational datasets, often characterized by incomplete interactions or categorical variables like knowledge components or skill identifiers, can be modeled directly by LightGBM without the need for complex preprocessing. This feature ensures the integrity of the underlying data and simplifies the modeling pipeline, making it particularly effective for PFA and DAS3H, where sparse and irregular data are common (Chen and Guestrin, 2016).

The model’s flexibility and efficiency extend to its regularization techniques, which control complexity and mitigate overfitting. LightGBM optimizes an objective function that includes both loss and regularization terms. For regression tasks, the objective function is:

$$\Theta =\sum_{i=1}^{M}L(y_{i},\hat{y_{i}})+\sum_{j=1}^{J}\Omega (f_{j})$$

Where L represents the loss function and Ω is the regularization term controlling tree complexity.

For classification, LightGBM employs binary cross-entropy loss, which effectively handles binary and multi-class problems:

$$\Theta =\sum_{i=1}^{M}[y_{i}\log(\sigma (\hat{y_{i}}))+(1-y_{i})\log(1-\sigma (\hat{y_{i}}))]+\sum_{j=1}^{J}\Omega (f_{j})$$

Where σ is the sigmoid function converting raw scores into probabilities.

These optimizations allow LightGBM to model complex interactions and dependencies across datasets, as seen in the holistic modeling required by DAS3H (Gervet et al., 2020; Ke et al., 2017).

While XGBoost has traditionally been a dominant framework in gradient boosting, recent studies emphasize LightGBM’s superiority in scenarios involving high-dimensional and sparse data. In IRT, where latent traits and item difficulties are central, LightGBM’s faster convergence and optimization efficiency offer tangible benefits. Similarly, in PFA, LightGBM’s capacity to handle evolving knowledge states dynamically outperforms static methods, providing deeper insights into student learning processes.

Given the efficiency and scalability of LightGBM, this study employs the framework to analyze seven real-world educational datasets. The methodology section outlines the datasets, preprocessing methodologies, and evaluation metrics used to benchmark LightGBM’s performance in predicting learner outcomes.

## 5 Explainability in educational data mining: XAI and SHAP

Predictive models such as IRT, PFA, and DAS3H have advanced our understanding of learner behaviors by incorporating factors like item difficulty, skill mastery, and temporal learning dynamics. However, while these frameworks are interpretable due to their explicit mathematical structures, modern machine learning models, such as LightGBM, often outperform them in predictive accuracy but at the cost of reduced interpretability. This “black-box” nature of LightGBM poses a challenge in educational settings, where actionable insights and transparency are vital. Explainable AI (XAI) methods, particularly SHapley Additive exPlanations (SHAP), address this challenge by quantifying the contribution of individual features to predictions (Lundberg and Lee, 2017). SHAP leverages a game-theoretic foundation to provide feature importance at both global and local levels, making it ideal for understanding how user-level, item-level, and skill-level features contribute to predictions.

In this study, SHAP is employed to interpret LightGBM’s predictions and to analyze the impact of diverse features across datasets, including user-level, item-level, and skill-level characteristics. By leveraging SHAP, this study evaluates how specific features such as temporal decay parameters, skill mastery and performance metrics, and item difficulty interact with other predictors in LightGBM to influence student performance predictions.

Integrating SHAP into this analysis bridges the gap between the interpretability of traditional models like IRT, PFA, and DAS3H, and the predictive power of machine learning. By quantifying the contributions of these features, SHAP offers actionable insights for educators and system designers, enabling targeted interventions and the refinement of adaptive learning systems. This approach aligns with the growing emphasis on transparent AI in education, as emphasized in recent studies (Molnar, 2019; Ribeiro et al., 2016).

## 6 Methodology

This study aims to comprehensively evaluate the performance of LightGBM in predicting learner outcomes using seven diverse real-world educational datasets. The methodological framework, illustrated in Figure 1, consists of several key stages: dataset selection, data preparation, feature engineering, model training, and evaluation. Each stage follows a structured approach to address the unique complexities of educational data while leveraging LightGBM’s advanced capabilities.

**Figure 1:**
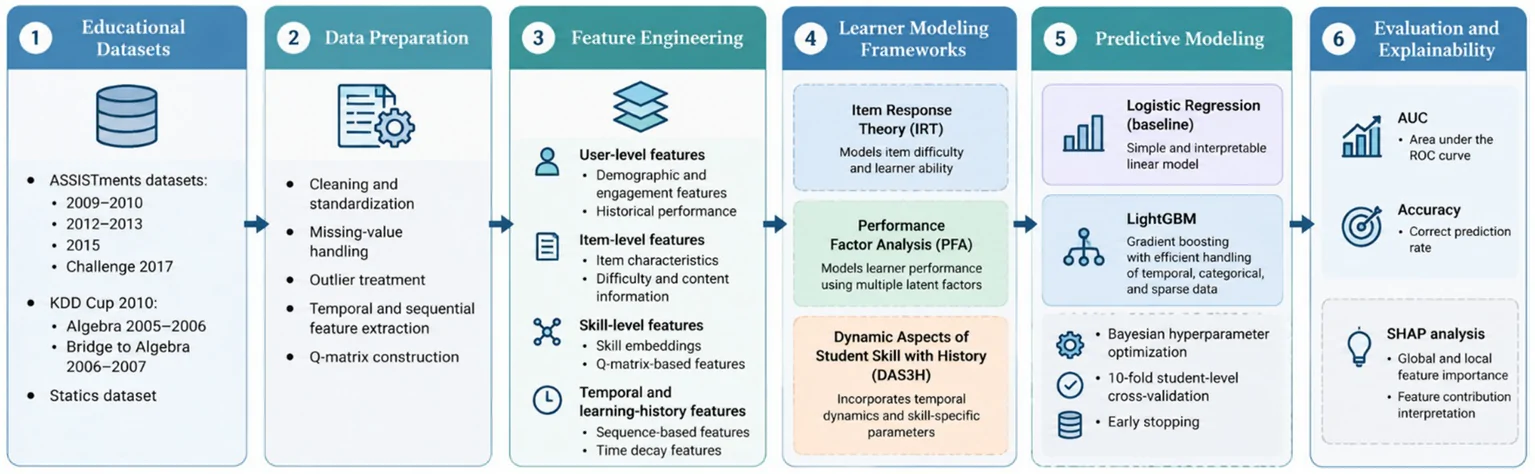
LightGBM prediction framework.

### 6.1 Datasets

To capture the diverse dimensions of educational learning, we selected seven publicly available datasets that span different domains and contexts. These datasets offer a rich variety of interactions, learning trajectories, and skill mappings, providing a strong foundation for predictive modeling.

#### 6.1.1 ASSISTments intelligent tutoring system

Four datasets (2009–2010, 2012–2013, 2015, and Challenge 2017) documenting student interactions, knowledge acquisition, and performance metrics in tutoring systems (Feng et al., 2009).

#### 6.1.2 KDD cup challenge 2010

Two datasets (Algebra 2005–2006 and Bridge Algebra 2006–2007) known for their temporal interaction data, ideal for modeling sequential learning behaviors (Stamper et al., 2010).

#### 6.1.3 Statics dataset (PSLC Datashop)

This dataset emphasizes problem-solving in engineering, featuring unique skill mappings and learning trajectories (Koedinger et al., 2010).

These datasets encompass various educational activities such as mathematical reasoning, problem-solving, and programming, making them suitable for assessing the robustness of predictive techniques.

### 6.2 Data preparation

Efficient data preparation is critical for ensuring the quality and relevance of features used in modeling. The preparation process consisted of three main components:

### 6.3 Cleaning and standardization

Data cleaning involved handling missing values and outliers to maintain dataset integrity. Missing values were imputed with statistical measures (e.g., mean or median) for less significant features, while entries with excessive missing data were excluded. Outliers were addressed through statistical techniques like z-scores or the interquartile range (IQR), and appropriate transformations ensured they did not skew results. Standardization steps unified data formats by normalizing categorical variables and scaling numerical features, promoting consistency across datasets.

### 6.4 Temporal and sequential feature extraction

To capture the dynamics of learning, temporal features were derived, including time gaps between interactions, rolling averages of performance, and time buckets. Sequence filtering retained students with adequate interactions, focusing on meaningful patterns in learning trajectories. These temporal and sequential attributes allowed the analysis of engagement trends and skill acquisition over time.

### 6.5 Q-matrix construction

A sparse Q-matrix was constructed to map the relationships between skills and questions, providing a structured representation of student performance. Each row in the matrix corresponds to a question, and each column represents a skill or knowledge component (KC). A binary value of 1 indicates that a particular question involves a given skill. This mapping enhances interpretability and facilitates efficient computation in conjunction with predictive models like PFA and DAS3H.

### 6.6 Feature engineering

In addition to existing features, new features listed in Table 1 were derived from each dataset to capture additional dimensions of student performance. These features were carefully extracted to enrich the modeling process while maintaining alignment with the specific characteristics of each dataset.

**Table 1:** Extracted features.

Dataset	Feature type	Features
ASSISTments	User-Level	- F1: Number of attempts per student - F2: Average correctness rate - F3: Time spent per attempt
	Item-Level	- F4: Difficulty level of each question - F5: Frequency of questions attempted by multiple students - F6: Time gap between consecutive attempts on the same question
	Skill-Level	- F7: Mastery level of knowledge components (KCs) (PFA and DAS3H counters) - F8: Temporal decay parameters for skill mastery (e.g., time since last practice) - F9: Aggregated success rates for specific KCs
KDD Cup	User-Level	- F1: Rolling averages of performance across sessions - F2: Engagement metrics (e.g., number of interactions per session) - F3: Prior knowledge estimates from IRT models
	Item-Level	- F4: Categorization of problems (e.g., algebra vs. geometry) - F5: Skill tag distribution for each item - F6: Log-transformed difficulty indicators
	Skill-Level	- F7: Success and failure counts with logarithmic scaling (PFA features) - F8: Question response patterns based on similar items - F9: Time-bucketed correctness ratios (DAS3H features)
Statics	User-Level	- F1: Total attempts and their outcomes - F2: Correctness ratio over time - F3: Clustering of errors based on similar question types
	Item-Level	- F4: Unique question identifiers and skill mappings - F5: Problem-solving steps required for each question - F6: Temporal factors such as time-to-solve and last activity timestamp
	Skill-Level	- F7: Aggregated performance on KCs tagged to problems - F8: Decay features modeling forgetting rates - F9: Student-specific latent ability traits (IRT-based)

By combining temporal, sequential, and Q-matrix-driven features, the datasets were enriched to comprehensively represent learner behavior. This approach ensured computational efficiency by avoiding extensive preprocessing, such as one-hot encoding, while leveraging LightGBM’s ability to natively handle categorical and sparse data. Feature engineering tailored the datasets for LightGBM by extracting and synthesizing meaningful predictors, incorporating Q-matrix-driven features alongside temporal and sequential attributes to create a robust and comprehensive representation of learner behaviors.

The Q-matrix played a pivotal role in this process, enriching the datasets by supporting dynamic frameworks like DAS3H. It enabled the derivation of essential counters, such as time-windowed successes, attempts, and skill decay, which are critical for modeling forgetting and practice effects. These enriched features seamlessly integrated into LightGBM, capitalizing on its capacity to handle categorical splits and sparse data efficiently. This integration reduced feature dimensionality and ensured computational efficiency, further enhancing the model’s capacity to process high-dimensional educational data.

LightGBM’s tree-based framework effectively captured non-linear dependencies among features, such as KC performance, question difficulty, and time-based trends. Its inherent flexibility allowed for the modeling of complex skill interactions and patterns of knowledge acquisition over time, significantly enhancing its predictive performance. These capabilities offered deeper insights into student learning dynamics, ensuring both accuracy and interpretability in the predictive modeling process.

### 6.7 Model training

To optimize LightGBM’s predictive power, the training process was carefully configured and refined:

### 6.8 LightGBM configuration

The LightGBM model was implemented using its Python library, capitalizing on its gradient boosting framework and tree-based learners. Key settings included:

#### 6.8.1 Hyperparameter tuning

Bayesian optimization fine-tuned critical parameters like learning rate (0.01–0.1), number of leaves (30–50), and tree depth (5–15). Regularization (L1 and L2) was employed to control complexity and reduce overfitting.

#### 6.8.2 Cross-validation

A 10-fold cross-validation at the student level ensured generalizability by splitting training and testing data across different subsets of learners.

#### 6.8.3 Early stopping

Training ceased when performance improvements on validation data plateaued, minimizing overfitting and computational overhead.

### 6.9 Evaluation framework

To rigorously assess model performance, two evaluation metrics were employed, targeting classification tasks:

#### 6.9.1 Area under the ROC curve

Assessed the model’s ability to distinguish between positive and negative outcomes, balancing sensitivity and specificity.

#### 6.9.2 Accuracy

Measured the proportion of correctly predicted instances, offering a holistic view of model reliability.This methodological approach integrates advanced preprocessing, robust feature engineering, and rigorous evaluation. By leveraging LightGBM’s efficiency and interpretability, the study delivers actionable insights into the dynamics of student learning behaviors, paving the way for enhanced educational modeling techniques.

## 7 Results

This section presents a comparative analysis of the performance of the LightGBM and LR models across various datasets, utilizing three educational frameworks: Performance Factor Analysis PFA, IRT, and DAS3H. The evaluation metrics employed include the AUC and Accuracy. These results are contextualized with reference to prior studies, notably those by Yeung and Yeung (2018) and Gervet et al. (2020), to highlight LightGBM’s performance enhancements.

The comparative performance of LightGBM and logistic regression on the KDD Cup datasets is presented in Table 2

**Table 2:** KDD cup score results.

Dataset	Model	AUC	Accuracy
Algebra 05	IRT-LR	0.771	0.801
	IRT- LightGBM	0.778	0.870
	PFA-LR	0.744	0.788
	PFA- LightGBM	0.769	0.803
	DAS3H-LR	0.826	0.817
	DAS3H- LightGBM	0.834	0.829
Algebra 06	IRT-LR	0.747	0.839
	IRT- LightGBM	0.752	0.845
	PFA-LR	0.739	0.841
	PFA- LightGBM	0.762	0.852
	DAS3H-LR	0.790	0.849
	DAS3H- LightGBM	0.805	0.857

The corresponding performance results for the Statics dataset are summarized in Table 3.

**Table 3:** Statics score results.

Dataset	Model	AUC	Accuracy
Statics	IRT-LR	0.789	0.805
	IRT- LightGBM	0.791	0.808
	PFA-LR	0.691	0.775
	PFA- LightGBM	0.719	0.782

The results obtained across the ASSISTments datasets are reported in Table 4.

**Table 4:** ASSISTment score results.

Dataset	Model	AUC	Accuracy
Assist09	IRT-LR	0.692	0.684
	IRT- LightGBM	0.699	0.689
	PFA-LR	0.724	0.706
	PFA- LightGBM	0.738	0.717
Assist12	IRT-LR	0.702	0.721
	IRT- LightGBM	0.642	0.732
	PFA-LR	0.6701	0.714
	PFA- LightGBM	0.702	0.784
	DAS3H-LR	0.739	0.743
	DAS3H- LightGBM	0.715	0.690
Assist15	IRT-LR	0.638	0.726
	IRT- LightGBM	0.642	0.732
	PFA-LR	0.690	0.739
	PFA- LightGBM	0.702	0.748
Assist17	IRT-LR	0.681	0.666
	IRT- LightGBM	0.688	0.673
	PFA-LR	0.620	0.647
	PFA- LightGBM	0.638	0.661
	DAS3H-LR	0.6932	0.677
	DAS3H- LightGBM	0.709	0.689

The PFA model, which captures both successful and unsuccessful attempts to dynamically assess skill progression, showed that LightGBM consistently outperformed logistic regression across all datasets. For instance, in the Algebra 05 dataset, LightGBM achieved an AUC of 0.769 compared to 0.744 for logistic regression, reflecting an improvement of +0.025. Similarly, in the Algebra 06 dataset, the AUC increased from 0.739 with logistic regression to 0.762 with LightGBM, highlighting its ability to capture nonlinear dependencies. These findings are consistent with those of Yeung and Yeung (2018), who emphasized the importance of using advanced models to capture complex learning trajectories. Moreover, improvements in accuracy were observed across the PFA comparisons, with gains ranging from modest increases to a notable +0.070 in the ASSIST12 dataset

In contrast, the IRT framework showed relatively comparable performance between LightGBM and logistic regression, with only modest improvements in most datasets. For example, in the Algebra 05 dataset, logistic regression achieved an AUC of 0.771, whereas LightGBM achieved 0.778. Similarly, in the Algebra 06 dataset, the AUC increased slightly from 0.747 with logistic regression to 0.752 with LightGBM. Comparable modest gains were also observed in ASSIST09, ASSIST15, and ASSIST17. However, this pattern was not consistent across all datasets. In ASSIST12, logistic regression achieved a higher AUC than LightGBM (0.702 vs. 0.642), although LightGBM achieved slightly higher accuracy (0.732 vs. 0.721). These findings suggest that the relatively simple structure of IRT may limit the additional benefit of a more complex nonlinear model such as LightGBM. Nevertheless, the results also indicate that LightGBM can provide modest improvements in some datasets by capturing nonlinear interactions between learner ability and item-related characteristics.

The DAS3H framework, which incorporates temporal dynamics and skill-specific learning histories, showed more variable benefits from LightGBM across datasets. In the Algebra 05 dataset, LightGBM achieved an AUC of 0.834 compared with 0.826 for logistic regression, representing an improvement of +0.008. Similarly, in Algebra 06, the AUC increased from 0.790 to 0.805, while in ASSIST17 it increased from 0.6932 to 0.709. These findings are consistent with the importance of temporal and sequential information in learner-performance modeling highlighted by Gervet et al. (2020). However, the advantage of LightGBM was not universal. In ASSIST12, logistic regression outperformed LightGBM in both AUC (0.739 vs. 0.715) and accuracy (0.743 vs. 0.690). Overall, these results indicate that LightGBM can effectively exploit the temporal and skill-dependent features represented in DAS3H, but its predictive advantage depends on the characteristics of the dataset. Greater model complexity therefore does not systematically guarantee better performance, emphasizing the importance of evaluating each model–framework combination across heterogeneous educational datasets.

The SHAP summary plots reveal critical insights into the strengths of LightGBM in predicting student performance by leveraging diverse feature sets. LightGBM’s advanced gradient-boosting approach allows it to effectively capture non-linear dependencies between user-level, item-level, and skill-level features, providing a competitive advantage over traditional models like logistic regression and static frameworks such as IRT. Unlike these simpler models, LightGBM’s leaf-wise tree growth strategy prioritizes splits with the highest loss reduction, enabling it to model complex, high-dimensional relationships in educational data (Ke et al., 2017).

The SHAP summary plots illustrating feature contributions across the ASSISTments, KDD Cup, and Statics datasets are presented in Figures 2–4, respectively.

**Figure 2:**
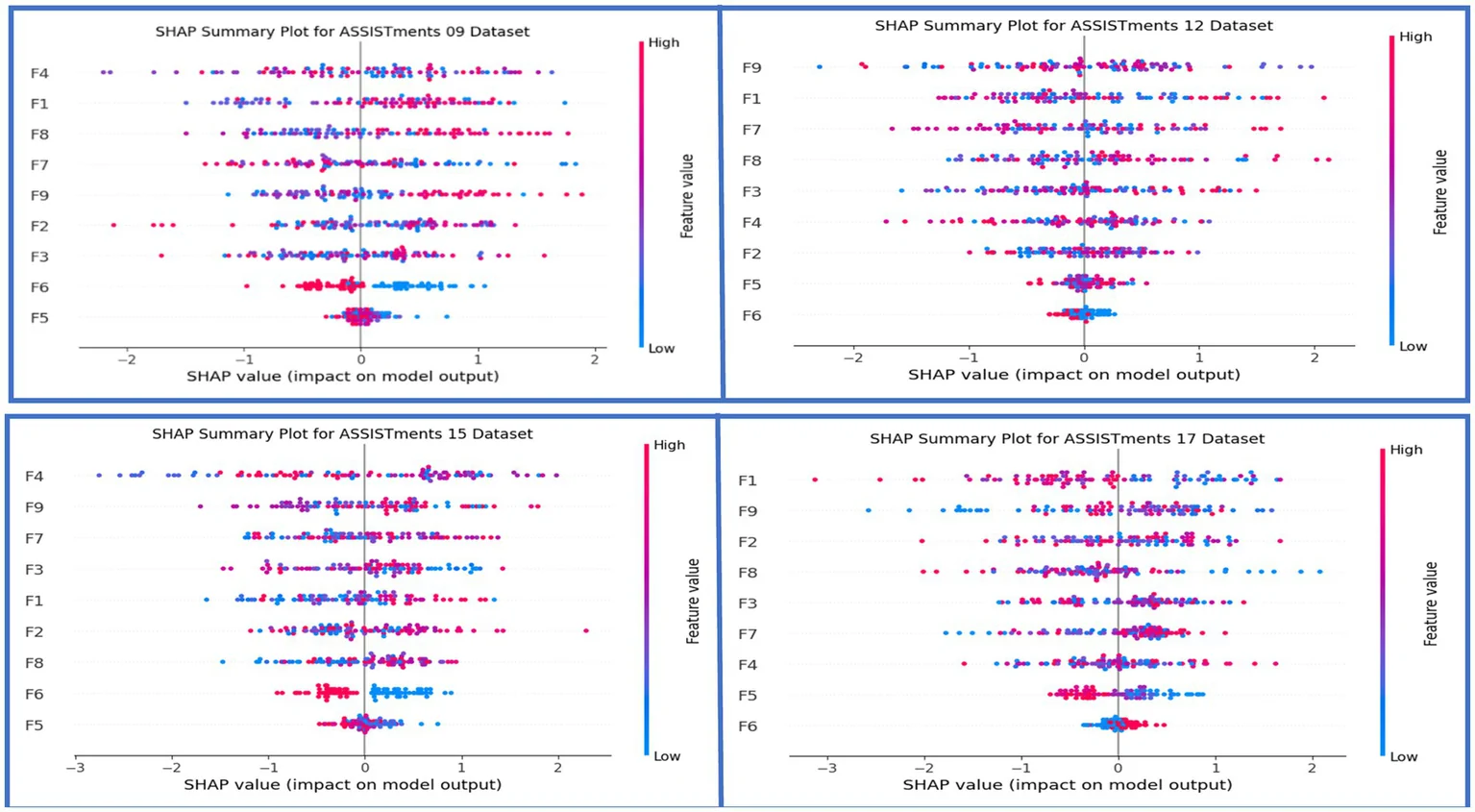
SHAP summary plots for feature contributions in ASSISTments datasets.

**Figure 3:**
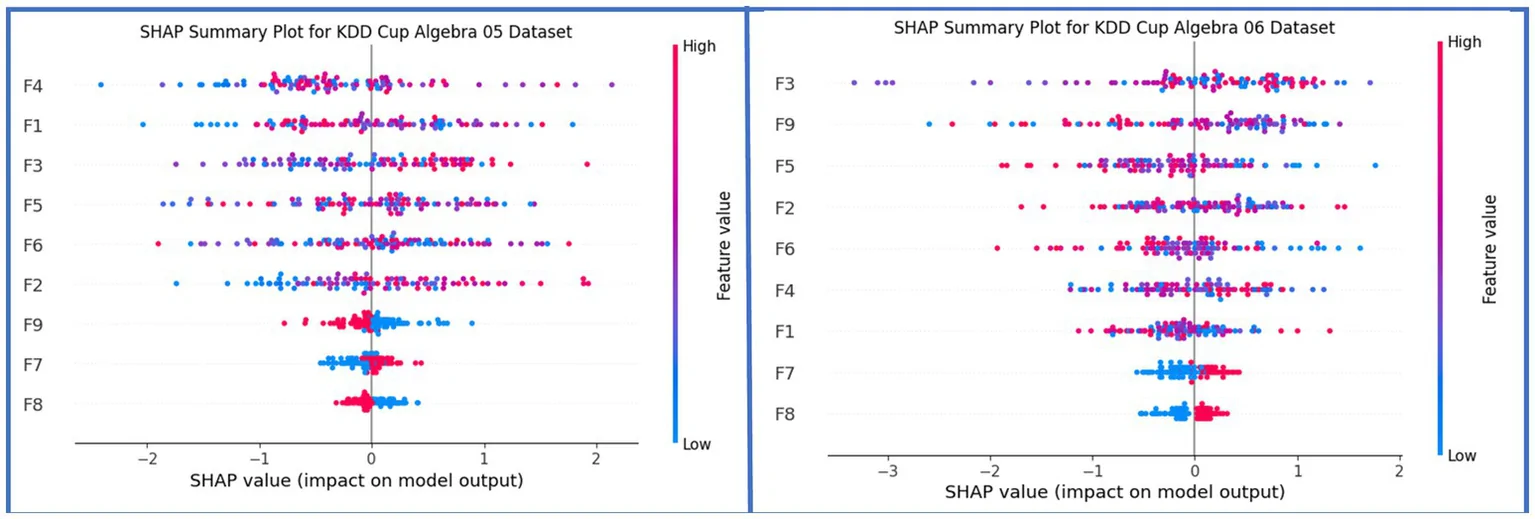
SHAP summary plots for feature contributions in KDD cup datasets.

**Figure 4:**
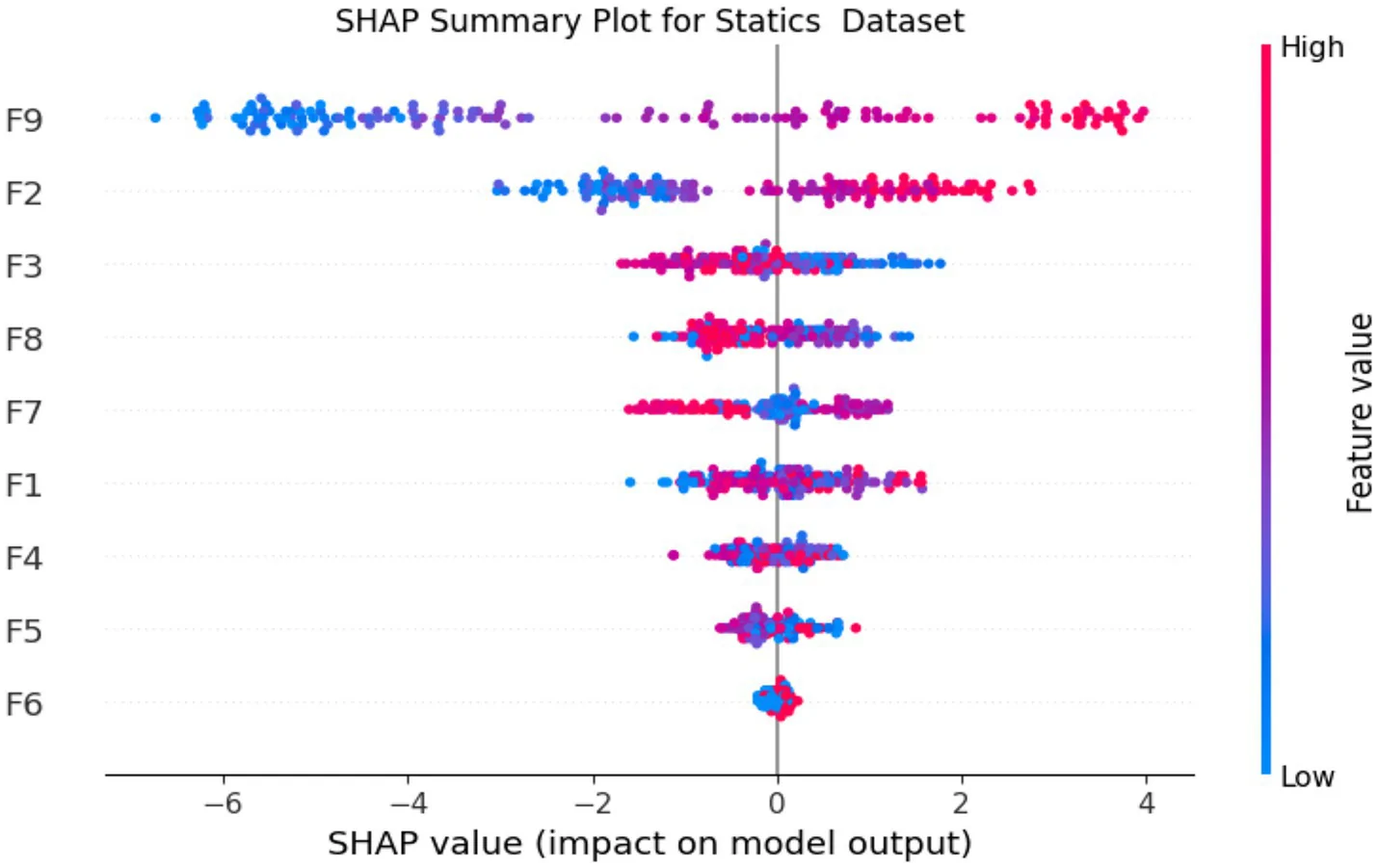
SHAP summary plots for feature contributions in statics dataset.

The SHAP analysis for the ASSISTments datasets in Figure 2, highlights LightGBM’s ability to identify key predictive features and their nuanced impacts across different datasets and learning models. For instance, user-level features such as the number of attempts per student (F1) and average correctness rate (F2) demonstrate high SHAP values, reflecting their critical importance in tracking engagement and skill mastery. LightGBM excels in capturing the interactions between these features and other temporal dynamics. For example, time spent per attempt (F3) exhibits moderate importance, but LightGBM identifies patterns that might not be apparent to linear models, such as how longer or shorter durations interact with difficulty levels and skill decay.

LightGBM’s strength in modeling item-level features is also evident in the SHAP plots, particularly for item difficulty (F4). This feature consistently shows high SHAP values across all datasets, underscoring the importance of tailoring predictions based on question complexity. Static models like IRT rely heavily on this feature but lack the flexibility to incorporate its interaction with temporal or cumulative features. LightGBM, on the other hand, integrates difficulty (F4) with features like time gap between attempts (F6) and mastery of knowledge components (F7), reflecting the layered complexity captured in PFA and DAS3H. The ability of LightGBM to combine these features into its decision trees highlights its versatility in handling multi-faceted datasets.

Skill-level features, particularly temporal decay parameters (F8) and aggregated success rates for knowledge components (F9), are another area where LightGBM outperforms traditional approaches in the Statics dataset. These features are critical in DAS3H, which incorporates time-windowed success and forgetting curves. The SHAP plots reveal that LightGBM assigns high importance to F8 and F9 in datasets with extensive student interaction histories, such as ASSISTments 12 and 17. This indicates that LightGBM effectively models the temporal decay of skills and the cumulative benefits of repeated successes, capturing both short-term learning gains and long-term retention patterns. These insights align with the principles of DAS3H, where temporal dynamics are explicitly accounted for, but LightGBM extends this by considering interactions with other features.

LightGBM’s flexibility is further enhanced by its native handling of missing values and categorical data, which are prevalent in educational datasets. This capability reduces the need for extensive preprocessing and allows LightGBM to directly leverage features such as problem categorization (F4) and skill tag distributions (F5). The SHAP analysis demonstrates that these item-level features play a significant role in predicting student outcomes, particularly in datasets like KDD Cup Algebra 05 and 06, where problem diversity and skill mappings are key predictors. LightGBM captures the interplay between these features and engagement metrics, such as rolling averages of performance (F1) and time-bucketed correctness ratios (F9), providing a comprehensive view of student learning trajectories.

Unlike logistic regression or IRT, which are constrained by their linear assumptions, LightGBM dynamically adjusts to the non-linearities inherent in educational data. For example, in datasets like ASSISTments 15, where temporal decay (F8) interacts with engagement (F1) and question difficulty (F4), LightGBM’s SHAP values reveal its ability to identify subtle patterns that static models miss. This adaptability aligns with prior findings that gradient boosting frameworks like LightGBM excel in capturing sequential learning behaviors and skill acquisition over time (Gervet et al., 2020).

Overall, the SHAP analysis showcases LightGBM’s transformative potential in educational data mining. Its capacity to integrate the strengths of IRT, PFA, and DAS3H while addressing their limitations makes it a powerful tool for understanding student performance. By leveraging SHAP, educators and researchers can interpret LightGBM’s predictions, uncover actionable insights, and design targeted interventions. The consistent high SHAP values across key features like F1, F4, F7, and F9 underscore LightGBM’s robustness in modeling diverse datasets, while its ability to incorporate temporal and cumulative aspects demonstrates its suitability for dynamic educational contexts.

These findings highlight the adaptability and robustness of LightGBM in capturing intricate learning patterns, as evidenced by its superior performance in the PFA and DAS3H models. The consistent improvements in AUC and ACC validate the advantage of employing advanced machine learning techniques in educational data modeling, further supporting the arguments of Yeung and Yeung (2018) and Gervet et al. (2020). However, the comparable performance of logistic regression in the IRT framework demonstrates the continued relevance of simpler models in scenarios with lower complexity. By integrating these results with prior research, it becomes evident that LightGBM’s ability to excel in dynamic and complex educational scenarios underscores its transformative potential for predicting and understanding learner performance. These results emphasize the importance of tailoring model selection to the specific characteristics of the dataset and the educational context, ensuring the appropriate balance between predictive accuracy and interpretability.

## 8 Implications of LightGBM for educational data mining

The findings of this study underline the transformative potential of advanced machine learning models like LightGBM in educational data mining. LightGBM generally achieves improved predictive performance compared with traditional logistic regression, particularly within the PFA framework and in several DAS3H settings, although performance gains vary across datasets. This enhanced predictive accuracy highlights LightGBM’s capacity to handle complex datasets with non-linear interactions and temporal dependencies, making it an invaluable tool for learner performance prediction in educational settings.

One of the key implications of LightGBM’s superior performance lies in its application to adaptive learning systems. Accurate and reliable predictions are essential for personalized education, as they enable systems to provide timely and customized feedback to learners. By modeling dynamic behaviors, LightGBM can improve student engagement and educational outcomes, paving the way for more effective and tailored learning experiences.

LightGBM’s ability to capture temporal decay and cumulative skill mastery provides a distinct advantage in frameworks like PFA, which require tracking learners’ histories to predict performance effectively. Unlike static models such as IRT, which assume a fixed learner ability, LightGBM accommodates evolving learning trajectories, offering more practical and realistic predictions. This dynamic capability positions LightGBM as a vital component in the development of modern educational analytics tools.

Another significant contribution of this study is the integration of SHAP analysis, which bridges the gap between predictive power and interpretability. By identifying the contributions of key features such as engagement metrics, question difficulty, and temporal learning trends, SHAP enables educators and system designers to make data-driven decisions. This level of explainability not only enhances the usability of LightGBM in practice but also supports targeted interventions and the refinement of teaching strategies.

The study also highlights the trade-offs between advanced machine learning models and simpler approaches. While logistic regression remains effective in static scenarios like IRT, LightGBM’s dynamic capabilities make it better suited for real-world, evolving educational contexts where learners’ abilities and engagement change over time. This insight underscores the importance of tailoring model selection to the specific requirements of the educational framework and data characteristics. Moreover, the consistent performance of LightGBM across diverse datasets, including ASSISTments, KDD Cup and Statics, demonstrates its scalability and reliability. Its ability to handle sparse, high-dimensional data makes it a versatile solution for a variety of educational settings, ensuring generalizability across different contexts and applications.

Finally, this study advocates for further exploration of learning rate optimizations and boosting techniques to enhance convergence speed and overall model performance. While LightGBM offers significant advantages, the increased computational complexity, particularly with large datasets, suggests the need for efficient training pipelines. Future research should focus on balancing these trade-offs to optimize the practical application of LightGBM in educational data mining.

In summary, LightGBM has the potential to revolutionize educational analytics by enabling more accurate predictions and interpretable insights. Its capabilities can drive innovations in curriculum design, adaptive learning systems, and policy-making, ultimately contributing to enhanced educational equity and outcomes.

## 9 Future studies

Future research should extend the proposed framework by exploring more advanced machine learning and hybrid predictive approaches capable of modeling the complex, nonlinear, and temporal nature of learner behavior. Recent studies in other complex domains demonstrate the potential of machine learning to integrate heterogeneous variables, identify latent patterns, and support accurate forecasting and decision-making. For example, machine learning has been successfully applied to renewable-energy forecasting and clustering for smart-grid planning (Şan and Şenyiğit, 2026), traffic-volume prediction using socioeconomic and infrastructure variables (Ekici et al., 2026), and temporally aligned financial forecasting (Balcıoğlu and Merter, 2026). Similarly, AI-driven predictive models have been investigated for dynamic network optimization and smart-building energy management (Jiang et al., 2026; Kayiran, 2026b), while fuzzy AI-based decision frameworks have demonstrated their usefulness in prioritizing multiple interacting factors under uncertainty (Badi and Bouraima, 2026).

Building on these developments, future studies in learner performance prediction could investigate hybrid architectures combining LightGBM with deep learning, reinforcement learning, clustering, or fuzzy decision-making methods. Particular attention should be given to temporally aligned validation, multimodal learner data, automated feature selection, and real-time updating of learner profiles. Future work should also evaluate these approaches across additional educational contexts and institutions while preserving explainability through methods such as SHAP. Such developments could improve the ability of predictive systems to capture evolving learner trajectories and support more timely, personalized, and interpretable educational interventions.

## 10 Conclusion

Predicting learner performance is critical for advancing educational quality and adapting intelligent tutoring systems to individual learners needs. This study highlights the superior performance of LightGBM in modeling learner behaviors, achieving generally improved predictive performance compared with traditional logistic regression, particularly within PFA and several DAS3H settings. LightGBM’s ability to handle complex, high-dimensional, and temporal data demonstrates its transformative potential in educational data mining.

Additionally, this study integrates XAI methods, particularly SHAP analysis, to bridge the gap between predictive accuracy and interpretability. By identifying key feature contributions, such as engagement metrics, question difficulty, and temporal learning trends, SHAP enhances the usability of LightGBM in educational contexts. This level of transparency supports data-driven decision-making and enables targeted interventions, providing actionable insights for educators and system designers.

Despite its strengths, LightGBM still requires refinement to optimize its performance in simpler frameworks like IRT, where logistic regression occasionally shows competitive results. Enhanced hyperparameter tuning, efficient feature encoding, and optimized training pipelines can further unlock LightGBM’s potential. Addressing computational efficiency will also be vital for its application to large-scale educational datasets.

By integrating advanced predictive capabilities with actionable insights through XAI, LightGBM offers a powerful tool for improving learner outcomes. Future research should focus on overcoming current limitations and expanding its applicability in real-world educational systems, paving the way for more personalized, adaptive, and effective learning experiences.

## Data Availability

The original contributions presented in the study are included in the article/supplementary material, further inquiries can be directed to the corresponding author/s.
